# Brain concentrations and brain–blood ratios of amitriptyline and nortriptyline in forensic postmortem cases

**DOI:** 10.1093/jat/bkaf017

**Published:** 2025-02-27

**Authors:** Mikayla Zoë van der Meer, Brian Schou Rasmussen, Michael Nedahl, Marie Katrine Klose Nielsen

**Affiliations:** Department of Forensic Medicine, Section of Forensic Chemistry, Faculty of Health and Medical Sciences, University of Copenhagen, Copenhagen, Denmark; Department of Forensic Medicine, Section of Forensic Chemistry, Faculty of Health and Medical Sciences, University of Copenhagen, Copenhagen, Denmark; Department of Forensic Medicine, Section of Forensic Chemistry, Faculty of Health and Medical Sciences, University of Copenhagen, Copenhagen, Denmark; Department of Forensic Medicine, Section of Forensic Chemistry, Faculty of Health and Medical Sciences, University of Copenhagen, Copenhagen, Denmark

## Abstract

Concentrations of amitriptyline and nortriptyline in postmortem blood samples may not accurately reflect the concentrations at the time of death due to postmortem redistribution or degradation. The brain is suggested as an alternative matrix since it is less subjected to postmortem redistribution and more protected against trauma and putrefaction, but reference concentrations in brain tissue are scarce. In this study, we aimed to provide concentrations in brain tissue and brain–blood ratios in 53 postmortem cases, where amitriptyline and/or nortriptyline were detected. To establish reference levels, each case was assigned to one of three classes according to the cause of death: (i) lethal intoxication by the sum of amitriptyline and nortriptyline or nortriptyline alone, (ii) lethal intoxication by the drugs in combination with other drugs, and (iii) the cause of death was not influenced by amitriptyline and/or nortriptyline. A positive correlation between blood and brain concentrations was found with a Spearman coefficient of 0.98. In 42 cases, where both drugs were detected, the 10–90 percentiles in brain tissue ranged from 0.17–9.1 mg/kg (median: 0.78 mg/kg) for amitriptyline and 0.22–5.0 mg/kg (median: 1.43 mg/kg) for nortriptyline across all classes. In 11 cases where only nortriptyline was detected, the percentiles ranged from 0.32–7.2 mg/kg (median: 0.28 mg/kg) in brain tissue. A median brain–blood ratio of 3.4 was found for amitriptyline, 8.5 for nortriptyline as a metabolite of amitriptyline and 9.7 for nortriptyline as an individual ingested drug. No significant difference was found between the different classes. The obtained brain concentrations and brain–blood ratio can contribute to the alternative or complementary use of brain tissue for future toxicological investigations.

## INTRODUCTION

Globally, it is estimated that 5% of the adult population suffers from depression, ranking it among the most common mental disorders [1]. The primary medical treatment for depressive disorder is antidepressant medication, including amitriptyline and nortriptyline [2]. Beyond their primary use in treating depressive disorders, these antidepressants find application in the management of mental disorders, insomnia, and pain treatment [3, 4]. They can cause a lethal intoxication in overdose, and fatal outcomes are more frequent compared to newer antidepressant medications [5]. Despite their declining use, these drugs remain relevant in postmortem toxicological examinations. The obtained postmortem drug concentrations in blood are compared to reference concentration intervals in blood in the non-toxic and toxic range to assess the contribution to the cause of death by the drug [6]. Amitriptyline and nortriptyline are known for their extensive postmortem redistribution, the alteration of drug levels occurring after death [7–9]. This can make it difficult to differentiate between non-toxic and toxic cases, potentially leading to an incomplete or inaccurate conclusion regarding the involvement of the drug [6, 10, 11].

It is therefore preferable to have an alternative matrix to determine the cause of death, especially in cases where pronounced postmortem redistribution is suspected or when no blood is available. Brain tissue is proposed as a suitable alternative, since the brain is often available, even when blood is absent due to trauma. In addition, the brain is less affected by postmortem redistribution and is better protected against putrefaction caused by invading microorganisms [10, 12]. Brain tissue can be useful in cases concerning psychoactive drugs like amitriptyline and nortriptyline since they target the central nervous system in the brain. However, establishing reference concentration intervals is crucial to distinguish therapeutic from toxic and lethal concentrations. At present, reference brain concentrations of amitriptyline and nortriptyline and brain–blood ratios are scarce, and only a few studies have reported brain concentrations from a small number of cases [13–19]. Therefore, we retrospectively examined postmortem cases where amitriptyline and nortriptyline were detected to provide concentrations in brain tissue and brain–blood ratios to contribute to the alternative or complementary use of brain tissue for future toxicological evaluation. Additionally, we investigated the variations between metabolite-parent drug ratios of amitriptyline and nortriptyline in blood and brain tissue across therapeutic, toxic, and lethal concentrations.

## Material and methods

### Case selection and classification

The determination of amitriptyline and nortriptyline in blood and brain was part of the routine toxicological investigation of autopsy cases at the Section of Forensic Chemistry, Department of Forensic Medicine, Copenhagen. Cases, where either amitriptyline and/or nortriptyline was quantified in blood and brain tissue, between 2016 and 2023 were selected, resulting in 53 cases. Information from those cases was extracted from the laboratory information management system (LIMS) and included blood and brain concentrations of the drugs and their contribution to the cause of death. Cases were divided into amitriptyline cases, where both amitriptyline and nortriptyline were detected, and nortriptyline cases, where only nortriptyline was detected. Since nortriptyline is an active metabolite, and amitriptyline and nortriptyline are considered to be equipotent **[20]**, the classification of the amitriptyline cases was based on the sum of the concentration of amitriptyline and nortriptyline.

The postmortem cases were distributed into three classes based on the compound concentration in blood and the cause of death registered in the LIMS system, which has taken the case circumstances into account. Those three classes were based on the classification system previously described by Reis et al. [21]. Cases attributed to class A had blood concentrations that were known to solely cause a lethal intoxication without any significant contribution of other drugs. Blood concentrations of the cases in class B were known to have a lethal outcome in combination with other pharmaceuticals, drugs of abuse, alcohol, or disease and blood concentrations and case circumstances of the cases in class C indicated that the compound was not involved in the cause of death.

### Samples and analytical procedure

Autopsies were typically performed within 4 days after arrival at the Department of Forensic Medicine; in the meantime, the bodies were stored at 5°C. Femoral blood was taken without ligation of the iliac vein, and 4–8 ml of sample was preserved with 100 mg sodium fluoride and 22.5 mg of potassium oxalate. Brain samples (5–20 g) were collected from the frontal lobe. The autopsy samples were stored at −20°C until being subjected to standard toxicological analyses [22, 23]. Grey matter from the cerebral cortex of the frontal lobe was manually cut with a scalpel and 500 mg was homogenized (1:3) with water [24]. The biological samples were prepared using a fully automated solid-phase extraction setup, and the quantification was performed by ultra-high performance liquid chromatography–tandem mass spectrometry (UHPLC–MS/MS) as previously described [23, 24].

### Statistical analysis

The blood and brain concentrations in class A, B and C were presented as the non-parametrically determined median and 10–90 percentile intervals, as they were less affected by outliers.

The comparison of two variables was analyzed using the Mann–Witney U-test or Wilcoxon test (paired). A comparison of several variables was conducted using the Kruskal–Wallis test, followed by Wilcoxon tests between the different variables. The Spearman’s correlation coefficient (ρ) evaluated correlations between blood and brain concentrations. The significance level for correlation was set at ρ > 0.7 [25]. The statistical program R, version 3.4.1, was used to determine statistical parameters.

## Results and discussion

### Blood and brain concentrations

A total of 53 cases were included in this study, resulting in 42 amitriptyline cases, where nortriptyline was detected as a metabolite together with amitriptyline, and 11 nortriptyline cases, where only nortriptyline was detected. For amitriptyline cases, the 10–90 percentiles in femoral blood were 0.042–1.7 mg/kg (median: 0.26 mg/kg) for amitriptyline and 0.032–0.90 mg/kg (median: 0.14 mg/kg) for nortriptyline across all classes (Table 1). In brain tissue, the corresponding 10–90 percentiles were 0.17–9.1 mg/kg (median: 0.78 mg/kg) for amitriptyline and 0.22–5.0 mg/kg (median: 1.43 mg/kg) for nortriptyline, respectively.

**Table 1.** Concentrations of amitriptyline and nortriptyline in postmortem femoral blood and brain tissue presented as median values and 10–90 percentiles

			Amitriptyline conc. (mg/kg)		Nortriptyline conc. (mg/kg)		The sum of amitriptyline and nortriptyline conc. (mg/kg)	
	Class^a^	*n*	Median	10–90 percentiles	Median	10–90 percentiles	Median	10–90 percentiles
Amitriptyline cases								
Femoral blood	A	5	1.8	0.65–5.5	1.0	0.37–1.9	3.6	1.0–7.0
	B	6	0.58	0.31–3.5	0.43	0.35–0.59	1.0	0.66–4.1
	C	31	0.15	0.033–0.48	0.10	0.030–0.42	0.23	0.076–1.0
	Total	42	0.26	0.042–1.7	0.14	0.032–0.90	0.39	0.085–2.2
Brain tissue	A	5	9.3	3.4–28	5.0	2.7–17	19	6.2–40
	B	6	2.1	1.0–14	2.7	2.5–4.1	4.5	3.7–18
	C	31	0.39	0.16–1.5	0.82	0.19–3.7	1.1	0.49–5.7
	Total	42	0.78	0.17–9.1	1.43	0.22–5.0	2.3	0.53–18
Nortriptyline cases								
Femoral blood	C	11			0.28	0.042–1.0		
Brain tissue	C	11			3	0.32–7.2		

a Class A: Cases where amitriptyline and/or nortriptyline solely caused a lethal intoxication. Class B: Cases where amitriptyline and/or nortriptyline contributed to the cause of death. Class C: Cases, where either amitriptyline or nortriptyline was involved in the cause of death.

For the 42 amitriptyline cases, the sum concentration of amitriptyline and nortriptyline was considered unrelated to the cause of death in 31 cases (class C). In six cases, the drugs were considered to contribute to the cause of death (class B) and in five cases, the drugs were considered to solely cause a lethal intoxication (class A). Despite the overlap between the concentrations in the three classes, the concentrations of amitriptyline, nortriptyline, and the sum of amitriptyline and nortriptyline showed a significant difference between the classes in both blood and brain tissue (*P* ≤ .001).

For the 11 nortriptyline cases, where no amitriptyline was detected, only cases with non-toxic levels of nortriptyline (class C) were found with 10–90 percentiles of 0.042–1.0 mg/kg (median: 0.28 mg/kg) in femoral blood and 0.32–7.2 mg/kg (median: 3 mg/kg) in brain tissue. No significant difference was found between the concentration in nortriptyline cases and the sum of amitriptyline and nortriptyline (class C) in both blood and brain tissue.

### Comparison with existing literature

For the amitriptyline cases, the femoral blood concentrations of amitriptyline in the three classes were comparable with the intervals provided by Söderberg et al. [26] for 459 cases divided into 159 A cases, 175 B cases, and 125 C cases (Table 2). Concentrations of nortriptyline as a metabolite were comparable with the intervals provided by Reis et al. [21] for 272 cases divided into 132 A cases, 100 B cases, and 40 C cases (Table 2). Concentrations in our study for the nortriptyline cases align with the concentrations found by Reis et al. [21], for 11 C cases with a median concentration of 0.28 and 10–90 intervals of 0.1 to 0.7 mg/kg.

**Table 2.** Concentrations of amitriptyline and nortriptyline in postmortem blood and brain tissue reported in the literature for amitriptyline cases

		Amitriptyline conc. (mg/kg)			Nortriptyline conc. (mg/kg)			The sum of amitriptyline and nortriptyline conc. (mg/kg)			
Matrix	Class	*n*	Mean	Range	*n*	Mean	Range	*n*	Mean	Range	Ref.
Femoral blood	A	159^a^	2.4^b^	0.9–6.9^c^							[26]
		138	2.5^b^	1–6.7^c^	132	0.9^b^	0.2–2.5^c^				[21]
		9	4.6	0.6–11	9	1.4	0.05–5.4	9	6.0	1.1–16	[13]
		4	3.7^d^	2.7–4.7^d^	4	1.1^d^	0.5–1.7^d^				[14]
		1	0.82^d^		1	1.1^d^					[15]
		1	7.0^d^		1	7.4^d^					[16]
	B	175^a^	1.6^b^	0.6–5.5							[26]
		119	1.7^b^	0.7–5.8^c^	100	0.4^b^	0.1–1.2^c^				[21]
		12	1.6	0.1–8.9	12	0.6	0.1–1.7	12	2.2	0.2–11	[13]
		1	2.9		1	2.8					[17]
		1	2.3^e^		1	0.88^e^					[18]
	C	125^1^	0.2^2^	0.1–0.4^3^							[26]
		88	0.2^b^	0.1–0.4^b^	40	0.04^a^	0.008–0.1^b^				[21]
	Unknown	2604	0.4^b^	0.1–2.6^f^	1347	0.3^b^	0.1–1.3^f^				[31]
		53	0.13	0.002–2.3	47	0.064	0.002–1.5				[32]
		14	2.7	0.14–8.7	14	1.1	0.09–3.4				[19]
Brain tissue	A	7	42	8.3–68	7	26	7.0–47	7	68	30–110	[13]
		4	11	2.6–18	4	3.9	0–7.7				[14]
		1	4.8		1	5.5					[15]
		1	29		1	24					[16]
	B	8	9.3	1.4–24	8	9.2	1.5–38	8	19	3.8–51	[13]
		1	8.7		1	15					[17]
		1	24		1	9.2					[18]
	Unknown	14	36	0.33–168	14	16	0.62–50				[19]

Class A: Amitriptyline solely caused a lethal intoxication. Class B: Amitriptyline contributed to the cause of death. Class C: amitriptyline was not involved in the cause of death.

a Includes cases by Reis et al. with additional cases.

b Median.

c 10–90 percentile.

d Blood source unknown.

e Iliac blood.

f LOQ–90th percentile.

In brain tissue, our median values of the toxic and lethal concentrations of amitriptyline, nortriptyline and the sum of the two drugs were 3.4–5.2 times lower than the mean concentrations reported by Kristinsson et al. [13]. This may be due to their mean being adversely affected by cases with very high concentrations, as their maximum reported values were also far greater than ours for both A and B cases in brain and blood. Contrary to this, lethal concentrations of amitriptyline and nortriptyline were reported in four cases by Baselt, which were consistent with our brain concentrations for A cases [14]. In another study, with the highest brain concentrations found in the literature, Prouty and Anderson reported a 46-fold higher mean brain concentration for amitriptyline (mean: 36 mg/kg, range: 0.33–168 mg/kg) and an 11-fold higher brain concentration for nortriptyline (mean: 16 mg/ kg, range: 0.62–50 mg/kg) compared to our median [19]. However, in this study by Prouty and Anderson, the contribution of the drugs to the cause of death was not disclosed, which complicated the comparison between the found brain concentrations. Since different concentrations may occur in different parts of the brain, comparisons between studies should be made with caution if the brain sampling site and atomical location are not known. This was clarified by Wille et al., who examined the concentrations of antidepressants in different parts of the brain including the temporal lobe, parietal lobe, occipital lobe, frontal lobe, brain stem, and cerebellum, and found varying concentrations between different compartments of the brain [27]. To aid future forensic investigations, it is therefore important that the sampling site is specified when establishing reference concentrations in brain tissue.

### Brain–blood ratio

**Figure 1.**
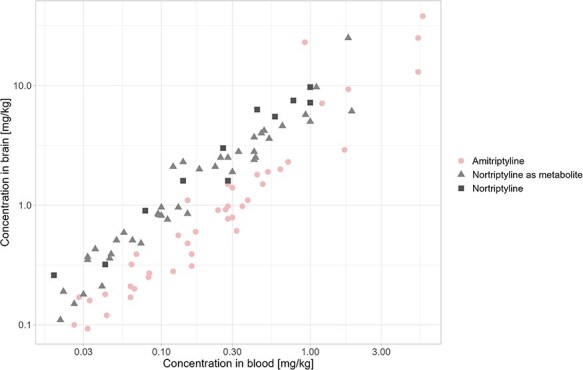
Relationship between the concentrations of amitriptyline and nortriptyline in blood and brain tissue. The graph shows the concentrations of 42 cases with amitriptyline and nortriptyline as a metabolite and 11 cases with nortriptyline in both blood and brain tissue on a logarithmic scale. The Spearman coefficient for the amitriptyline cases was 0.96 for amitriptyline and 0.98 for its metabolite nortriptyline. A Spearman coefficient of 0.96 was found for the nortriptyline cases, indicating a strong correlation between blood and brain concentrations.

In general, the drug concentration was higher in the brain than in the blood, and a positive correlation was observed between amitriptyline concentration in blood and brain tissue (ρ: 0.96). The same observation was made for the concentrations of nortriptyline in blood and brain tissue, both in the presence of amitriptyline (ρ: 0.98) and without (ρ: 0.96) (Fig. 1). This indicates that brain tissue could be a suitable alternative in cases where no blood is available or when blood is not suitable for analysis.

**Figure 2.**
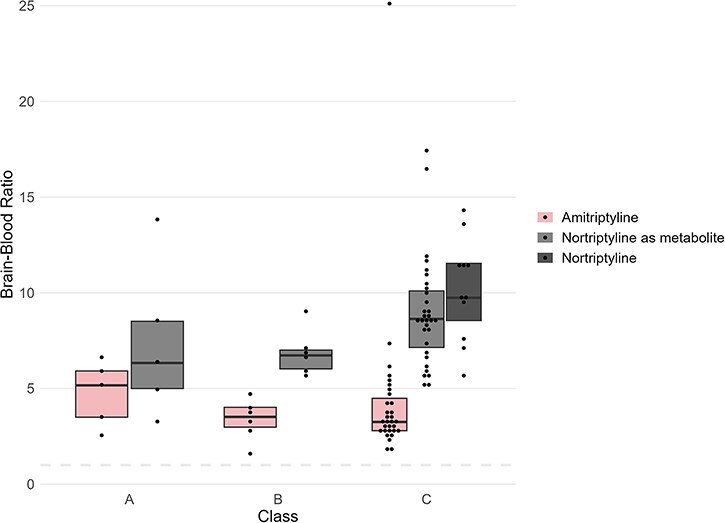
Brain–blood ratios of amitriptyline and nortriptyline in postmortem cases. The graph shows the ratios of 42 cases with amitriptyline and nortriptyline as a metabolite and 11 cases with nortriptyline. Cases are divided into three classes: Class A (5 cases) includes cases where the antidepressants were the cause of death, class B (6 cases) includes cases where the antidepressants were involved in the cause of death and class C (31 cases) where the antidepressants were not involved. The dashed line represents a ratio of 1, indicating that the concentration in brain tissue is the same as in femoral blood.

For the amitriptyline cases, median brain–blood ratios were 3.4 for amitriptyline, 8.5 for nortriptyline, and 5.5 for the sum of amitriptyline and nortriptyline. Upon classification, the median brain–blood ratios obtained for amitriptyline were 5.2, 3.5, and 3.3 in classes A, B, and C, respectively. For nortriptyline, the median brain–blood ratios were 6.3, 6.7, and 8.6 in classes A, B, and C, respectively. No significant difference was found between classes for either amitriptyline or nortriptyline indicating that the brain–blood ratio was independent of whether the drug was measured in therapeutic, toxic, or lethal concentration. The obtained brain–blood ratios for each class were plotted in Fig. 2.

As shown in Fig. 2, an overlap between the brain–blood ratio of nortriptyline was observed for nontoxic cases (class C) in amitriptyline and nortriptyline cases. This suggests that the brain–blood ratios are independent of whether the occurrence of nortriptyline originates as a metabolite from amitriptyline intake or as an administrated drug. Comparable conclusions were reached for morphine in a previous study when examining 69 morphine cases (morphine brain–blood ratios: 0.15–10.3) and 29 heroin cases (morphine brain–blood ratios: 0.31–7.9) where morphine was detected as a metabolite of heroin [28].

### Metabolite-parent ratio

In the 42 amitriptyline cases, a higher median metabolite-parent ratio of 1.4 was obtained in brain tissue compared to a median metabolite-parent ratio of 0.66 in femoral blood, which is similar to earlier published median metabolite-parent ratios of 0.99 in brain tissue and 0.67 in femoral blood [19]. The lower observed metabolite-parent ratio in blood compared to the brain could in part be explained by amitriptyline being more amenable to postmortem redistribution compared to nortriptyline. This would increase the concentration of amitriptyline in blood further than for nortriptyline. The brain, being a stable matrix, would be less susceptible to this effect [10]. Both the distribution of substances in brain tissue antemortem and the degree of postmortem redistribution may depend on the molecules’ physiochemical and pharmacokinetic properties, such as volume of distribution (Vd), ionisation state, lipophilicity, and molecular size [29, 30]. The structural difference between amitriptyline and nortriptyline is only a single methyl group while most of the molecule remains unchanged. However, this change is enough for their properties to differ.

**Figure 3.**
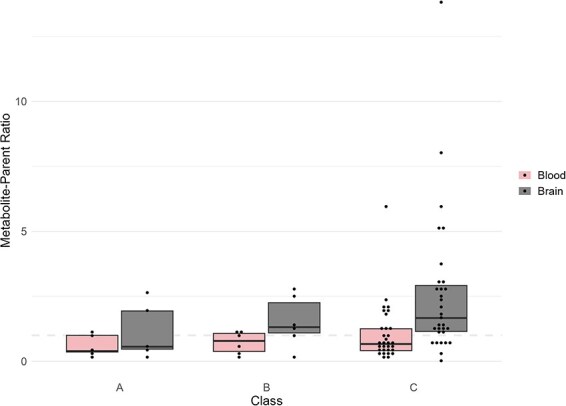
Metabolite-parent ratio of amitriptyline and nortriptyline in blood and brain tissue in the 42 amitriptyline cases. The graph shows the ratios in blood and brain tissue across class A, B, and C. Class A includes cases where the antidepressants were the cause of death, Class B includes cases where the antidepressants were involved in the cause of death, and Class C includes cases where the antidepressants were not involved. The dashed line represents a ratio of 1, indicating that the concentration of amitriptyline is the same as nortriptyline.

Upon classification, the median metabolite-parent ratio in blood was 0.39, 0.78, and 0.67 for classes A, B, and C, respectively. In brain tissue, the corresponding metabolite-parent ratio was 0.56, 1.3, and 1.8, respectively. Although there was a trend toward lower median metabolite-parent ratios in class A, an overlap of metabolite-parent ratios between classes was observed for both blood and brain tissue (Fig. 3).

## Conclusion

In this study, we provided therapeutic, toxic, and lethal concentrations of amitriptyline and nortriptyline in brain tissue obtained from 53 postmortem cases. Higher concentrations of both amitriptyline and nortriptyline were found in brain tissue compared to blood, and there was a positive correlation between brain and blood concentrations. Brain-blood ratios were established for amitriptyline (median: 3.4), nortriptyline (median: 8.5), and the sum of both drugs (median: 5.5) across all three classes. No significant deviation was observed between the classes, indicating that the brain–blood ratio is independent of the drug concentration. The comparison of nortriptyline in cases where only nortriptyline was present and cases, where both amitriptyline and nortriptyline were present, suggested that the brain-blood ratio is independent of the specific drug administered. The reported brain concentrations and brain-blood ratios can provide important information in future forensic investigations when using brain tissue as an alternative or complementary matrix.

## Data Availability

The data underlying this article cannot be shared publicly due to the nature of the study, which involves retrospective analysis of postmortem material collected from routine cases. Data sharing is limited to protect confidentiality and is per institutional guidelines.
